# A clearing in the objectivity of aesthetics?

**DOI:** 10.3389/fnimg.2023.1211801

**Published:** 2023-08-15

**Authors:** Daniel H. Lee, Junichi Chikazoe

**Affiliations:** ARAYA Inc., Tokyo, Japan

**Keywords:** deep neural networks, principal gradient, transmodal, neuroimaging, aesthetics

## Abstract

As subjective experiences go, beauty matters. Although aesthetics has long been a topic of study, research in this area has not resulted in a level of interest and progress commensurate with its import. Here, we briefly discuss two recent advances, one computational and one neuroscientific, and their pertinence to aesthetic processing. First, we hypothesize that deep neural networks provide the capacity to model representations essential to aesthetic experiences. Second, we highlight the principal gradient as an axis of information processing that is potentially key to examining where and how aesthetic processing takes place in the brain. In concert with established neuroimaging tools, we suggest that these advances may cultivate a new frontier in the understanding of our aesthetic experiences.

## Introduction

Aesthetics is messy. It is partial to our varieties of taste. Hume (1757) noted that while, in general, people agree about beauty, “united in applauding elegance, propriety, simplicity, spirit in writing… [when it comes] to particulars, this seeming unanimity vanishes”. His essay continues to discuss how the opposite appears true in matters of science, where disputes are “oftener found to lie in generals than in particulars [and disputants are surprised to find that] an explanation of the terms commonly ends the controversy”. One might then infer from Hume's observations that the science of aesthetics is elevated to an even untidier plane. Deprived of a generalizable sample of aesthetic experiences, attempting to generalize a set of principles about them seems destined to result in disorder.

As messy as aesthetics may be, experiences of beauty matter. “Art” remains one half of the practicing pair opposite “Science”. We revere and preserve artifacts of beauty that move us, so understanding aesthetic experiences should be of interest to anyone interested in understanding human behavior. We can also preclude a familiar criticism that studying aesthetic experiences tarnishes them by noting that a science of aesthetics is not itself an art. Similarly, we need not abandon the field of psychology because it is populated by adequate psychologists who conduct human affairs inadequately.^1^

Indeed, aesthetics has been studied by philosophers and neuroscientists alike (e.g., Hume, 1757; Zeki, 2001). Aided by functional magnetic resonance imaging (fMRI), neuroscientists have contributed to the modern understanding of aesthetics by, for example, showing the neural correlates of experiences of beauty (O'Doherty et al., 2003; Zeki et al., 2014; Vessel et al., 2019) and modeling neural representations of visual properties associated with these experiences (Iigaya et al., 2023). Furthermore, recent studies have found aesthetic appreciation to be one of the many dimensional representations in emotional responses to naturalistic stimuli (i.e., video) during fMRI (Horikawa et al., 2020; Koide-Majima et al., 2020). However, the thicket of aesthetic problems remains dense. How aesthetic experiences arise in the brain is a mystery, let alone why they do, and what rules govern their appraisal. Here, we draw attention to a pair of recent advances whose concerted application with neuroimaging may help hew a clearing in our understanding of aesthetic experiences. We elaborate upon them in brief.

## Deep neural networks

The popularization of deep learning artificial neural networks (ANNs) (LeCun et al., 2015) has spilled into the mainstream (OpenAI, 2023). At a slower but steady drip, ANNs and machine learning have been observed to have increasing applications in neuroimaging (Kell and McDermott, 2019). ANNs have even been employed in our domain of concern, modeling subjective and aesthetic value in the brain (Kragel et al., 2019; Iigaya et al., 2023).

The broad utility of deep ANNs lies in their capacity to universally map any input to any output (Hornik et al., 1989). Therefore, speaking in general, theoretical terms, we could argue for their fitness to model and test any representations of stimulus to value, including aesthetic ones. More acutely, however, ANNs may be tailored to address a vital problem of aesthetics, the problem of form. Let us illustrate by way of a concrete example from Huxley, who writes of a character in “Crome Yellow” (Huxley, 1921) pleading about the value of a certain arrangement of words:

*I proffer the constatation, “Black ladders lack bladders.” A self-evident truth, one on which it would not have been worth while to insist, had I chosen to formulate it in such words as “Black fire-escapes have no bladders”…. But since I put it as I do, “Black ladders lack bladders,” it becomes, for all its self-evidence, significant, unforgettable, moving. The creation by word-power of something out of nothing–what is that but magic?*

**Figure 1:**
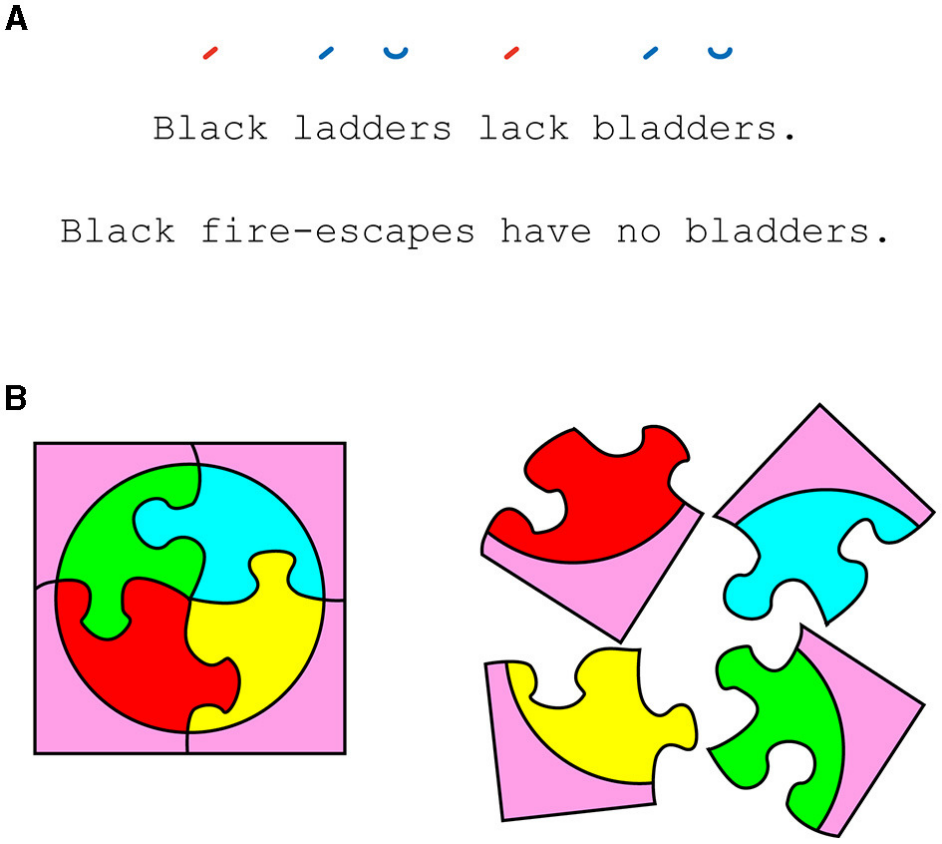
**(A)** An aesthetic illusion. Both statements convey the same content but differ in value when read. Scansion notation (top) represents the form responsible for the greater value: matching rhymes (colored) and meter (stressed–stressed–unstressed feet). **(B)** The same four-piece features of a jigsaw puzzle are on the left and right. The relations between the pieces affect how we value them as a whole.

What Huxley's character professes as magic, a psychologist could call a value illusion, specifically of an aesthetic variety. Then, deconstructing the illusion as adequate psychologists, we find the self-evident meanings of both statements controlled, such that the additional value of the first statement must be conferred by its form. Specifically responsible are its two synchronous phonetic pairs, one in rhyme and one in rhythm (i.e., meter) (Figure 1A). In other words, the additional meaning is delivered by an implicit property of the stimulus rather than its explicit content.

We have many such notions of implicit stimulus properties: style, structure, texture, relations, arrangement, pattern, music, cadence, tone, and so on. For simplicity, we subsume these under “form”. Form plays an important role in art. Artists misapprehend it at the peril of being branded literal. In contrast, empirical science appears to be allergic to the implicit. For example, while Behaviorism (Watson, 1913) ended up sweeping Wundtian (Wundt, 1897) and Jamesian (James, 1890) concerns of apperceptions, relations, and the implicit under the rug of the subjective, a decades-long movement was underway in the arts, whereby painters appeared to be navigating earlier and earlier into the visual cortex, away from things toward forms (e.g., Impressionism, Minimalism, and Suprematism).

Form is elusive. Its implicit character is but one reason for science's prejudice. Another reason is that describing form can seemingly destroy it. However, this is mere appearance, causal in nature rather than some romantic defense of the arts.^2^ More to the point, no matter the elusiveness of form, a science of aesthetics will require grappling with and submitting to it sufficient attention—not to stir the aesthetic within but to understand what, why, and how it stirs. Viewing Huxley's illusion through an engineering lens may be clarifying. Imagine you were tasked with coding an algorithm that could preferentially value the verse above the prose. Your algorithm would not only consider semantics but also represent the character and relations between phonemes.

The argument for deep ANNs is that they provide the architectural means to represent form in a way previously unavailable. This is because deep ANNs model not just the rudimentary features of stimuli but also non-linear relations between the features that are lacking in, say, traditional general linear models. Further, the “depth” of deep ANNs refers to hierarchical levels of latent information, across which features and their relations are functionally integrated into abstractions, becoming new features for further relations and abstraction (LeCun et al., 2015).

The capacity for ANNs to encode complex forms has been tangibly demonstrated, ranging from the representation and transfer of the style of Van Gogh (Gatys et al., 2016; Ramesh et al., 2022) to that of GPT-4's (OpenAI, 2023) convincing mimicry of Tarantino-sequel dialogue. However, modeling form is only part of the equation. It would behoove the inquiring, scientific mind to test which forms are valued and how the brain appraises them to generate an aesthetic value. However, we cannot yet nakedly model the brain as an ANN. A galaxy of atoms and floating-point operations stand in our way. For the time being, we are in need of guidance as to where and how in the brain our aesthetic processing takes place.

## Principal gradient

The second advance we highlight is the principal gradient (PG). A recent survey has suggested the PG to be a fertile tract for encoding a hierarchy of information from external stimulus to subjective values (Margulies et al., 2016). It accounts for the greatest variance in resting-state functional connectivity. Its arrangement begins from multiple satellites of unimodal sensory information and then converges transmodally toward its terminus, the default mode network (DMN). The DMN is an integrative network of regions heavily implicated in self-referential processing (Raichle, 2015) that incorporates abstract, domain-general value processing centers (Chikazoe et al., 2014). In short, the PG appears to encode an axis of information hierarchy from unimodal sensory information to amodal conceptual information that culminates at the seat of subjective information processing.

One way to appreciate the relevance of the PG to our aims is by noticing that aesthetic experiences arise as readily from what the subject's mind constructs as they do from artistic stimuli. If this is not immediately evident, it is in part because science is not the only party of prejudice, and our vernacular for aesthetic expectation is often monopolized by the arts. However, there is no material reason that our aesthetic operations on conceptual, modeless stimuli should be of different assembly than those operating on paint brushed by a master. Consider how aesthetic experiences can additively interact across modalities. Imagine the orbital waltz in Stanley Kubrick's *2001: A Space Odyssey* without “The Blue Danube,” its partnering score. Verse is prose plus music (Figure 1A). Such multimodal consonance is the aesthetic processing of an abstract, amoral kind. Or, more directly, consider the following example outside the arts.

Many scientists can attest to an experience of beauty by contemplating the theory of evolution—how a simple concept pairing genetic variation and natural selection seemingly snaps into order a vast array of emergent biological phenomena. If the reader can empathize with this anecdotal evidence, we submit the following: the first is that this can be an aesthetic experience rivaling any great work of art. Second, this experience is conceptually rather than sensorily contingent, demonstrating that aesthetic processing can take place on abstractions beyond our primary sense modalities. Third, the beauty of evolution does not arise from its mere idea, its scientific repute, or even a sorted catalog of the animal kingdom. It seems necessary for the mind to reflect some relations between fauna and flora, and their forms and functions predicated upon each other, aligning survival's fit of particulars into some collective whole with other fauna, flora, and clime (Darwin, 1859).

This is not unlike the completion of a grand jigsaw puzzle, where pieces alone are not sufficient to elicit a preferred value, leaving the relations between them as the explanatory variable (Figure 1B). In other words, even our higher-order concepts possess form, some of which can be preferred for aesthetic appraisal. Whether dealing with the concrete or the abstract, mental representation of such relations, though not sufficient, appears essential for beauty's arrival. While we can admit that the beauty of evolution may not commute across subjects as aptly as a Monet, we might also notice that what is missing across these subjects is more akin to data than an algorithm. Consider an adolescent scientist. She learns evolution from an elementary textbook. The grand theory fails to awe. Is this a deficit of inheritance in the girl's capacity for beauty? Unlikely. The answer is better chalked up to “a lack of experience,” and in time, she would gather the features and relations necessary to compute evolution's rightful value.

Vast is the neural landscape across which aesthetic value might be appraised, with layers ranging from sensory modalities to abstract concepts. For the explorer, the PG provides the important first sketches of where and in which direction to look. We note that the last stop of the PG, the DMN, has been found to correlate with aesthetic experiences in both visual (O'Doherty et al., 2003; Vessel et al., 2019) and conceptual domains (Zeki et al., 2014). These terminal regions associated with value experiences are significant (Horikawa et al., 2020). However, we should be sobered by the fact that we are not equally privy to the implicit influences in aesthetic processing. Understanding them will likely incorporate how they are constructed hierarchically (O'Doherty et al., 2021), from the forms of stimuli to the invisible layers of abstractions and their interactions that are conducted behind the curtain of experience. The PG relieves us from mapping the whole brain and frees our confinement from examining only the final regions coactive with experience by pointing to a tractable middle ground that may include the precursors necessary for aesthetic value.

## Integration

Once we know where to look, we can employ ANNs to test the hierarchy of neural information. When confined to the visual domain, large-scale comparisons of image-classification ANNs have shown a general trend in associating improved classification performance with a greater prediction of higher visuocortical activity (Schrimpf et al., 2020). Importantly, studies have shown that the hierarchy of information encoded across ANN layers corresponds to the hierarchy of information across the visual cortex (Güçlü and van Gerven, 2015) and have demonstrated this multiple times, including by bidirectionally encoding and decoding the hierarchy of ANN information using voxel activity and vice versa (Nonaka et al., 2021).

Expanding to the domain of value, we can train an ANN on a subject's aesthetic valuations and then examine which voxels along that subject's PG best correspond to that ANN. Then, treated as analogs, we can test whether the hierarchy of information in the ANN corresponds to the neural representations along the hierarchy of the PG. Such an early examination suggests such a relationship for the aesthetic processing of paintings by individuals (Pham et al., 2021).

It goes without saying that the true edge of complexity lies beyond our current reach. There remain difficult questions of which forms rise to aesthetic significance, why they rise, when they rise, and in what context. Subjectivity too is not just a problem across individuals but a moving target within, as our experiences subsequently influence how we value a thing once new grows habitual, old. Traumatic experiences can hammer what we value in one direction or another. The value of Darwin's contribution may feel more important as an adult once contextualized with the disappointing scope of most adult theories.

On a brighter note, if a single example here resonates, it may be counted as a signal of objectivity in our aesthetic experiences. We are fortified by these signals. A science of aesthetics need not be dismissed as sullying our exalted experiences of beauty. Rather than compartmentalizing an entire category of human experience as impenetrably divine, the study of aesthetics may be more profitably framed as lowly pastures in want of tilling. We may at present be in possession of the tools.

## Data availability statement

The original contributions presented in the study are included in the article/supplementary material, further inquiries can be directed to the corresponding authors.

## Author contributions

DHL and JC contributed to the design and provided the conception and overall guidance for the project. DHL contributed to the initial drafting of the manuscript. All authors contributed to the writing, revision, and approval of the manuscript.

## Footnotes

^1^A thing and its abstraction are distinct. Confounding the two is to commit a reference error.

^2^The implicit cannot be made explicit by definition. The implicit can be referenced by way of an explicit abstraction, but they are distinct. For example, the scansion notation in Figure 1A is an explicit representation of the underlying music, but the representation itself does not express the music.
